# Surgical Treatment of Spinal Deformities in Pediatric Orthopedic Patients

**DOI:** 10.3390/life13061341

**Published:** 2023-06-07

**Authors:** Sebastian Braun, Marco Brenneis, Lukas Schönnagel, Thomas Caffard, Panagiotis Diaremes

**Affiliations:** 1 Department of Orthopedics (Friedrichsheim), University Hospital Frankfurt, Goethe University, 60590 Frankfurt am Main, Germany; marco.brenneis@kgu.de (M.B.); panagiotis.diaremes@kgu.de (P.D.); 2 Stavros Niarchos Complex Joint Reconstruction Center, Hospital for Special Surgery, New York, NY 10021, USA; 3 Charité—Universitätsmedizin Berlin, Freie Universität Berlin and Humboldt-Universität zu Berlin, Center for Musculoskeletal Surgery, 10117 Berlin, Germany; lukas.schoennagel@charite.de; 4 Spine Care Institute, Hospital for Special Surgery, New York, NY 10021, USA; thomas.caffard@rku.de; 5 Department of Orthopaedic Surgery, University of Ulm, 89075 Ulm, Germany

**Keywords:** scoliosis, Scheuermann’s disease, spinal deformities, diagnosis, treatment, physiotherapy, bracing, surgical intervention, kyphosis, spinal curvature

## Abstract

Scoliosis and Scheuermann’s disease are common spinal deformities that affect a substantial population, particularly adolescents, often impacting their quality of life. This comprehensive review aims to present a detailed understanding of these conditions, their diagnosis, and various treatment strategies. Through an extensive exploration of current literature, the review discusses the etiology of these spinal deformities and the use of diagnostic tools such as X-rays and MRI. It further delves into the range of treatment options available, from conservative approaches such as physiotherapy and bracing to more invasive surgical interventions. The review underscores the necessity of an individualized treatment approach, taking into account factors such as the patient’s age, the severity of the curvature, and overall health. This all-encompassing perspective on scoliosis and Scheuermann’s disease will aid in evidence-based decision making in their management with the goal of improving patient outcomes.

## 1. Introduction

Spinal deformities are the most frequent forms of orthopedic deformity in children and adolescents. This review article addresses the topic of pediatric spinal deformities, which differ from those of adults in both incidence and characteristics. The focus is on some of the most common disorders of the pediatric spine such as scoliosis and Scheuermann’s disease (SD), providing a summary of each condition and outlining the typical treatment options that are part of the pediatric orthopedic armamentarium.

## 2. Methodology

In conducting this narrative review, we performed a comprehensive search of the PubMed database with the following key terms: ‘scoliosis’, ‘early onset scoliosis’, ‘Scheuermann’s disease’, ‘surgical treatment’, ‘spinal deformities’, ‘adolescent idiopathic scoliosis’, ‘kyphosis’, and ‘spondylodesis’. By using the Boolean operator “AND” in our search strategy, we were able to combine these terms in various unrestricted free-text searches to gather a broad spectrum of relevant literature. No temporal constraints were applied to our search; however, we directed particular focus toward the most recent publications in the field. Additionally, other reviews and original articles were sourced from the reference lists of the relevant papers identified, ensuring a thorough coverage of the topic, particularly with regard to surgical interventions. In conducting this narrative review, it is essential to acknowledge potential sources of bias, primarily originating from the fact that the literature selection and interpretation of study results were subjective and depended on the authors’ expertise and perspectives in the field.

## 3. Scoliosis

The treatment of scoliosis in childhood and adolescence is still very challenging. In addition to conservative options, there are various surgical therapies available for guiding growth, such as distraction-based methods including traditional growing rods (TGR), magnetically controlled growing rods (MCGR) and vertical expandable prosthetic titanium rib (VEPTR). Similarly, compression-based approaches such as vertebral body stapling (VBS) and vertebral body tethering (VBT), as well as growth-guiding procedures such as Shilla and Luqué trolley are possible treatment options. 

Scoliosis is defined as a structural curvature of the spine in the horizontal, frontal and sagittal planes with a Cobb angle of at least 10°. The curvature involves torsion of the vertebral bodies in the transverse plane. As a result of this curvature, structural changes may occur in the axial skeleton as well as in the thorax. Scoliosis occurs in children and adolescents up to the age of 16 years, with a prevalence of approximately 2% [1]. Scoliosis can be classified into two main groups: idiopathic type and secondary scoliosis. The idiopathic form comprises approximately 80–90% of all the types of scoliosis and is a diagnosis of exclusion of other forms of scoliosis. Despite advanced research studies, the primary etiology remains unknown. In addition to having an important genetic component, many other molecular, biochemical, neurologic, and environmental factors have been described. Idiopathic scoliosis can occur at different stages of life [2,3,4]: infantile scoliosis (0 to 3 years: ~1–5%), juvenile scoliosis (4 to 10 years: ~10–20%), and the most common form, adolescent scoliosis (>11 years: ~80–90%). Scoliosis that occurs at earlier ages may be associated with marked developmental and growth impairment [4,5]. The second main group includes secondary scoliosis associated with neuropathic (with central or peripheral motor neuron involvement or both), myopathic, or syndromic etiologies (e.g., Marfan syndrome, Ehlers–Danlos syndrome, neurofibromatosis, or other skeletal dysplasias) [6,7]. 

Early-onset scoliosis (EOS) summarized a myriad of conditions, which is united by the documentation of scoliosis in young children. There is controversy regarding the upper age limit for diagnosis, but a consensus among some authors is that it should be around 10 years old [8]. EOS includes spinal deformity resulting from neuromuscular conditions, from dysplasias and syndromes, from congenital malformations, and from idiopathic cases. The progression of EOS varies depending on its etiology, but the treatment remains challenging. If left untreated or managed through spinal fusion, which can result in a shorter trunk and spinal height, EOS can have serious health consequences, including increased morbidity and even mortality [9]. Table 1 shows possible differential diagnoses of scoliosis. 

### 3.1. Spinal Growth

Childhood growth can be divided into three phases. From birth until about the age of five, there is a rapid growth phase where the trunk and leg lengths increase equally [11]. During these years, the spine reaches about two-thirds of its later total length [12]. This rapid phase is followed by steady, slower growth where the leg length increases twice as much as the trunk length. Then, from around the age of 11 in girls and 13 in boys, there is a (pre)pubertal growth spurt (acceleration phase) where the trunk length doubles the increase in leg length [11]. The first sign of puberty is an increase in the growth speed to more than 0.5 cm per month or 6 cm per year [12]. The maximum growth speed in the lower extremities is reached six months before that of the spine [11]. Growth in the lower extremities ends when the overall growth speed peaks at the end of the acceleration phase, leaving about 4.5 cm of remaining trunk growth [12].

From birth until growth concludes, the spine from T1 to S1 grows approximately 25 cm, with nearly 40% of this growth occurring in the first five years. Between the ages of 5 and 10, growth reduces to 5 cm, and after the age of 10, another 10 cm of growth is expected. About 40% of the spine’s growth occurs after the age of 10 [3].

At two years of age, standing height is roughly 50% of adult height; at five years, it is about 60%, and by the age of nine, it is approximately 80%. The standing height comprises two specific measurements: subischial height (the growth of the lower limbs) and sitting height (the growth of the trunk) [13]. In patients with scoliosis, it can be beneficial to track changes in sitting height rather than standing height. The growth of the spine and its curvature during these phases can indicate the need for treatment. For instance, any spinal curve that increases by 1° each month during the ascending phase of the pubertal growth is likely to be progressive and will require treatment. Similarly, any curve that increases by less than 0.5° each month during this phase can be considered mild [13]. The risk of scoliosis evolution is proportional to growth and initial angulation. A curve of 20° at the onset of puberty has a risk of undergoing surgery of 16%, while a curve of 20° to 30° has a risk of 75%, and a curve of 30° has a risk of 100% [13]. These risks decrease as puberty progresses. Steadily progressing deformities of the spine in the early years of life pose considerable health risks for the growing child [14,15], such as permanent respiratory impairments due to impaired lung development [16,17].

The thorax’s development plays a pivotal role during puberty. From birth, where the thorax only makes up about 6% of the total body size, it sees a five-fold increase by age five and doubles again by age ten [13]. This significant thoracic growth during puberty is integral to understanding and treating scoliosis. The growth spurt not only accompanies the spinal deformity but can also exacerbate it. Moreover, the spinal deformity progression can have detrimental effects on lung development, specifically the alveoli, potentially leading to restrictive lung disease. This, in turn, can progress into pulmonary arterial hypertension, which is a key contributor to right heart failure or pulmonary heart disease [3,13]. This intertwined relationship between thoracic growth, restrictive lung disease, and spinal deformity is termed thoracic insufficiency syndrome, as proposed by Campbell and Smith [15]. Therefore, a comprehensive understanding of spinal growth principles and changes during different growth stages is crucial for managing conditions such as scoliosis and SD.

### 3.2. Diagnostics

The patients’ history should include potentially related conditions, such as congenital heart defects or urological and urogenital abnormalities, including a horseshoe kidney [18]. Additionally, the family history may provide clues about a hereditary predisposition to scoliosis. It is important to assess pain and mental distress through targeted questioning. Furthermore, it is advisable to inquire about the timing of the menarche, the rate of growth, and any current growth spurts that may be occurring [10].

In the clinical examination of the patient, postural anomalies or structural changes such as asymmetry of the waist triangles, shoulder elevation on the convex side, shoulder depression on the concave side, or lateral deviations of the procc. spinosi from the perpendicular are noticeable. Of particular importance is the Adams test, in which the patient bends forward with the legs extended. An existing leg length difference should be compensated beforehand. The test makes rib hump and lumbar bulge clearly visible. The supplementary measurement by means of a scoliometer according to Bunnel provides degree values which, multiplied by a conversion factor of 2.5–3, allow an assessment of the scoliosis even without radiological diagnostics (a measured value of 5 corresponds to an approximate Cobb angle of 15° in the X-ray image) [19,20].

**Figure 1:**
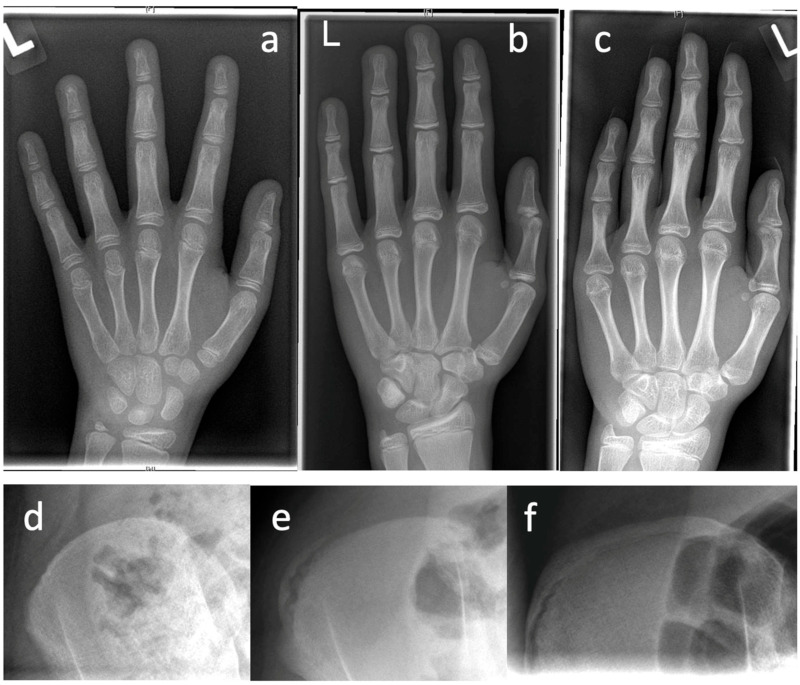
Examples for determination of growth potential according to Sanders of ne non-dominant left (L) hand [21]. (**a**–**c**) and Risser [22] (**d**–**f**), (**a**)—Sanders 2, (**b**)—Sanders 3, (**c**)—Sanders 5, (**d**)—Risser 0, (**e**)—Risser 2, (**f**)—Risser IV.

Table 2 presents diagnostic features that can be obtained in patient’s history, clinical examination, and in imaging procedures. Prior to initiation of therapy, determination of skeletal growth potential based on radiologically imaged skeletal maturity according to Risser and Sanders (Figure 1) etc. is of great importance.

A plain X-ray of the entire vertebral column, taken with the patient standing, is necessary. Ideally, this should include the iliac crests to evaluate bone growth status and the clavicles to observe shoulder stance. The severity of scoliosis is measured by the Cobb angle in the anterior–posterior X-ray view. This angle is calculated by identifying the two vertebral bodies most noticeably tilted from the horizontal, which is known as the end vertebrae [10]. The diagnosis of scoliosis also requires the presence of rotational deviation. This is indicated by the projection of the pedicles or procc. spinosi toward the concavity of the curvature in the anterior–posterior X-ray view. The lateral X-ray view provides information about the sagittal profile of the spine. In cases of sagittal deformities of the spine, compensatory rotation of the pelvis may occur, pivoting on the femoral heads to restore the spine to an upright position.

For certain subgroups of patients with a higher likelihood of spinal abnormalities, an MRI is recommended [3]. These subgroups include patients with atypical types of curvature (such as left convex thoracic), patients with symptomatic scoliosis, and individuals with abnormalities detected during neurological examinations. An MRI can provide valuable information regarding the presence of vertebral anomalies [3].

### 3.3. Conservative Therapy

Conservative treatment is indicated for idiopathic scoliosis with a Cobb angle of less than 45° in skeletally immature patients. For mild and moderate idiopathic scoliosis, conservative treatment options such as physical therapy (<25°) and additional bracing (25–45°) may be used to slow or stop the progression of the curvature. To treat scoliosis and other deformities conservatively, it is crucial to use individually made corrective orthoses as ready-made orthoses are not suitable due to the need for individual pressure point adjustments. Effective treatment requires an orthosis to be made individually based on plaster cast or laser 3D measurement which is followed by a radiological and pressure point control after a six-week adjustment period. In order to be effective, the orthosis must be worn at least 16–23 h a day. Clinically, it is crucial to prevent localized skin reactions such as redness, abrasions, deep lesions, and pain, as they reduce the compliance and time the orthosis is worn and thus compromise the treatment effect. Therefore, individual surface adjustments of the pads are necessary for orthopedic adaptation. The most common orthoses types for scoliosis are the Boston Brace, Lyon Brace, and Chêneau Brace, while hyperextension models with thoracic reclination pads are used for SD [24,25]. For skeletally immature patients with a Cobb angle ranging from 25° to 45°, bracing is recommended to slow down the progression of the curvature rather than decrease it, which should be discussed with the patient and parents.

In these cases, bracing should be continued until skeletal maturity and is most successful for a flexible deformity in those with a Risser stage of 0, 1, or 2 [26].

### 3.4. Surgical Therapy

#### 3.4.1. Indication for Surgery

Although many spinal deformities respond well to conservative therapy and do not progress or progress very slowly, particularly severe progressive courses or therapy failures at a young age require early surgical treatment [15,24]. In congenital disorders, the course may be marked and severe. In such cases, there is a risk of permanent inability to ambulate, impaired trunk posture and sitting ability, and restrictive ventilatory disorders due to an unstable thorax or severe thoracic deformities. To avoid respiratory insufficiency syndrome and allow further growth of the spine, surgical treatment by the most commonly used growth-guiding and distraction-based implants such as Growing Rods or VEPTR is indicated before definitive spondylodesis, which can be considered after the completion of spine growth [27]. In cases of progressive deformity despite conservative therapy, a growth-guiding surgical procedure is indicated to ensure further growth, particularly of the thoracic spine, resulting in increased lung volume.

**Figure 2:**
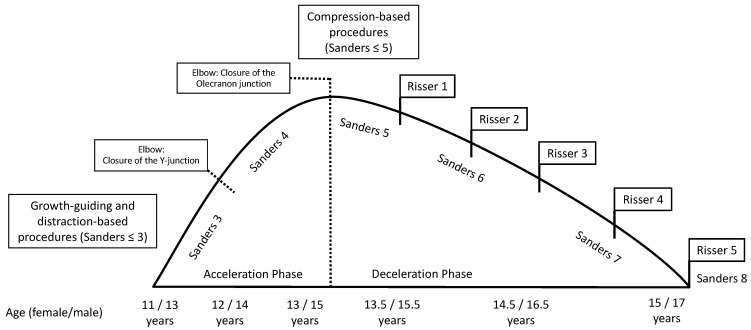
Therapy suggestions/indications and landmarks of pubertal growth featuring acceleration and deceleration phases according to Sanders, Sauvegrain (Elbow), and Risser (modified from [11,13]).

Von Deimling et al. recommend that the indication is based on the following criteria: curvature progression >10° or curvature >35° according to Cobb, RVAD (rib vertebral angle difference) >20° [28]. However, the Cobb angle criteria is not uncontroversial, which is also due to the fact that corset therapy has a substantial justification in this range. Cheung et al. therefore prefer to set the indication for surgery above 45–50° [29]. Figure 2 illustrates a suggested approach to scoliosis treatment in relation to skeletal age. Furthermore, surgery should not be performed until the age of 5 if possible. Consistent brace therapy or, in particular, serial plaster dressing may allow a gain in time up to this age. Regardless of the Cobb angle, pulmonary function decline, progression of scoliosis, conservative treatment failure (brace failure/non-compliance), and progressive loss of quality of life should be considered in the overall assessment and indication [28]. In systemic diseases with impending thoracic insufficiency syndrome, stabilizing and growth-guiding surgery should be considered much earlier [15].

In 2001, Lenke et al. developed a novel system for classifying spinal deformities [30]. Their goal was to devise a method using two-dimensional X-ray images that would simplify and enhance the reliability of categorizing all curvature types. It was also intended to factor in the sagittal profile and facilitate a standardized approach to surgical treatment. The Lenke classification system differentiates between structural and non-structural curvatures and organizes adolescent idiopathic scoliosis (AIS) into six curve patterns. These include main thoracic, double thoracic, double major, triple major, thoracolumbar/lumbar without a structural thoracic curve, and thoracolumbar/lumbar with a structural thoracic curve. A lumbar modifier is employed to denote the distance of the lumbar spine from the midline. Additionally, the sagittal profile is categorized as thoracic hypo-, normo-, or hyperkyphotic. The purpose of the Lenke classification is to provide clear criteria for each curve type that can guide the surgical treatment plan. Generally, surgical correction is recommended for structural curvatures, while non-structural curvatures may naturally correct over time.

#### 3.4.2. Preoperative Radiological Assessment

The standard imaging is the X-ray of the entire spine (posterior–anterior and lateral view) in standing position. With the EOS™ Imaging procedure, as an alternative to conventional X-rays, a three-dimensional reconstruction can be made using low-radiation, biplanar images of the spine [31]. With these images, the extent of spinal curvature in the frontal plane according to Cobb, the pattern of curvature in major and minor curvature, the sagittal profile or sagittal balance, the apical vertebra, the upper and lower neutral vertebra, the vertebral body rotation according to Nash/Moe to determine the rotational component, and the RVAD according to Mehta [32,33] are displayed. The RVAD can be used to assess the progression of the extent of curvature in scoliosis. Values > 20° are associated with a high probability of progression and should therefore be treated surgically [34].

Bending images are indicated before surgical interventions for planning the extent of instrumentation. In these images, the curvature type is classified according to Lenke, or the extent and corrigibility of the primary curvature and the compensatory counter-curvature are determined. Before planned surgery or in the case of neurological deficits and in order to exclude other spinal pathologies such as vertebra malformation or syringomyelia, it is critical to obtain a magnetic resonance imaging (MRI) scan of the entire spine, since intraspinal anomalies occur in 20–50% of these patients [3]. Formation and segmentation disorders can also trigger neurological symptoms.

#### 3.4.3. Growth Prognosis

Before initiating surgical therapy, knowledge of the patient’s growth potential is of immense importance, since without possible residual growth, some of the procedures cannot guarantee sufficient improvement of the extent of scoliosis. Spinal growth can be estimated on clinical and radiographic parameters. First, in girls, the onset of menarche is considered the point at which the pubertal growth spurt is complete and the growth tendency has already diminished. In most cases, the spine is fully grown about 2 years after the onset of menarche. In boys, the comparable counterpart is the change of voice. The most widespread radiographic method of determining the growth tendency and thus the ability of the spine to be corrected is based on the degree of ossification of the iliac apophysis according to Risser [22]. The iliac apophysis is divided into 6 stages according to different stages of ossification of the apophysis, which begins at the lateral iliac crest and progresses medially. A more reliable measurement is the Sanders classification (simplified Tanner–Whitehouse III system) using an X-ray of the non-dominant hand [21]. The state of ossification of the epiphyses of the hand and wrist defines the expected skeletal growth in 8 groups: juvenile slow (1), preadolescent slow (2), adolescent rapid (early) (3), adolescent rapid (late) (4), adolescent steady (early) (5), adolescent steady (late) (6), early mature (7), mature (8).

#### 3.4.4. Surgical Goals

Distraction-based (TGR, MCGR and VEPTR), compression-based (VBS, VBT) or growth-guiding (Shilla, Luqué trolley) procedures are used for surgical treatment. Table 3 provides a comparative overview of various techniques used in the treatment of scoliosis, highlighting their specific systems, indications, advantages, and disadvantages.

The goal of surgical correction using distraction-based growing rods is to achieve effective and balanced correction of all levels while preserving as many mobile spine segments as possible and avoiding neurological complications. Both ventral and dorsal surgical procedures can yield good results in terms of correction, functionality, and patient satisfaction when treating AIS. Ventral surgical procedures, in a single session, can typically address single-curve deformities (Lenke Type 1 or Type 5) [35]. The highest vertebra that can be reached via ventral surgery is approximately the fifth thoracic vertebra (T5) [36]. Dorsal correction has an advantage over ventral corrective spondylodesis in AIS because nearly all types of curvatures can be addressed dorsally. Double-curve and high-thoracic curvatures can generally only be corrected dorsally. Another limitation of ventral surgery is related to pulmonary function. Patients with significantly impaired lung function should not undergo ventral thoracic surgery due to the increased intraoperative and postoperative risk of further deterioration in lung function [37,38].

With distraction-based systems, the growth of unfused vertebral bodies is guided, and affected segments are continually derotated and guided to the correct position in the frontal and sagittal planes [39]. By growth guidance of the spine, a sufficient and best possible trunk height can be achieved to avoid dwarfism or body dysmetria with possible stigmatization. In recent years, there has been a significant increase in MCGR compared to TGR, VEPTR, or growth-guiding procedures such as Shilla and Luqué trolley procedures. Distraction-based MCGRs now represent over 80% of all implanted growth rods [40]. Pedicle screws are the standard in posterior scoliosis correction and are superior to hook systems. Sublaminar bands and wires have a similar potential for coronal correction as pedicle screws [41]. It is important to keep the desired fusion of the instrumented vertebral segments as short as possible to prevent spontaneous fusion of the adjacent segments due to periosteal irritation. In cases of stiffer AIS curvatures or pronounced sagittal deviation, Ponte osteotomies can be performed. However, there is no specific threshold of stiffness, Cobb angle, or sagittal profile that indicates when a Ponte osteotomy is recommended, so these decisions are made on a case-by-case basis [36].

### 3.5. Distraction-Based Techniques

#### 3.5.1. Traditional Growing Rods

**Figure 3:**
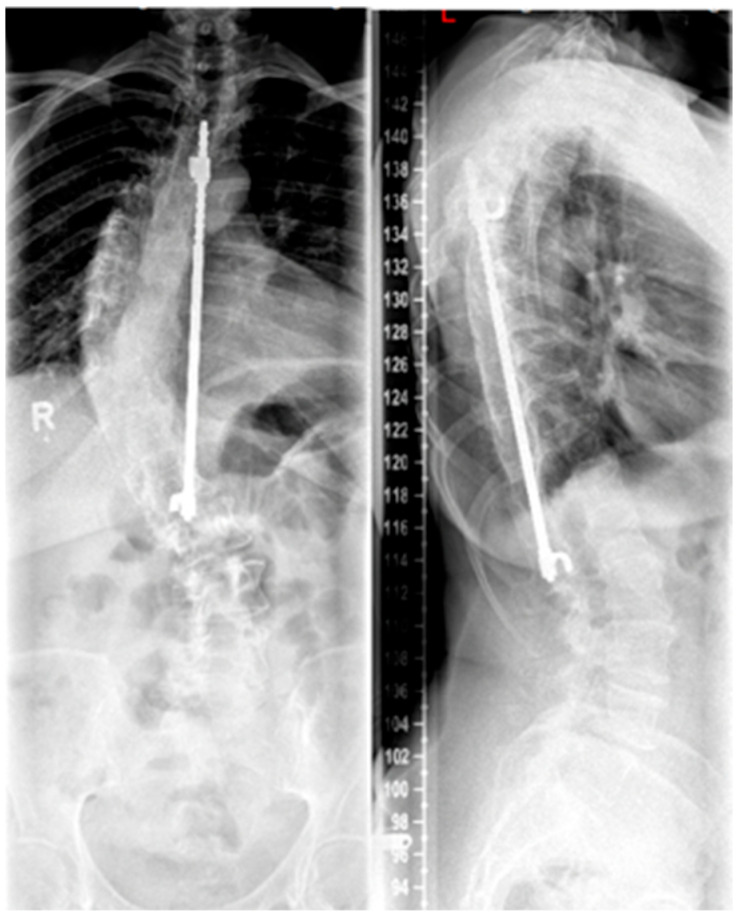
Patient post-Harrington instrumentation from T4 to L1 performed in 1980 (reprinted with permission [10]).

Growing rods are distraction-based systems that allow correction of the scoliotic spine in children and adolescents during growth. Since the first surgical techniques were described by Harrington in the 1960s (see Figure 3), with the goal of achieving spinal alignment by distraction without vertebral body fusion [42], there has been a significant evolution in the field of non-fusion techniques for scoliosis treatment. Because of this advancement, growing rods represent a standard procedure in the treatment of EOS. The idea behind TGRs is to straighten and realign the spine during growth by periodic lengthening of the instruments at least two times per year until completion of growth followed by definitive fusion [43].

#### 3.5.2. Vertical Expandable Prosthetic Titanium Rib

**Figure 4:**
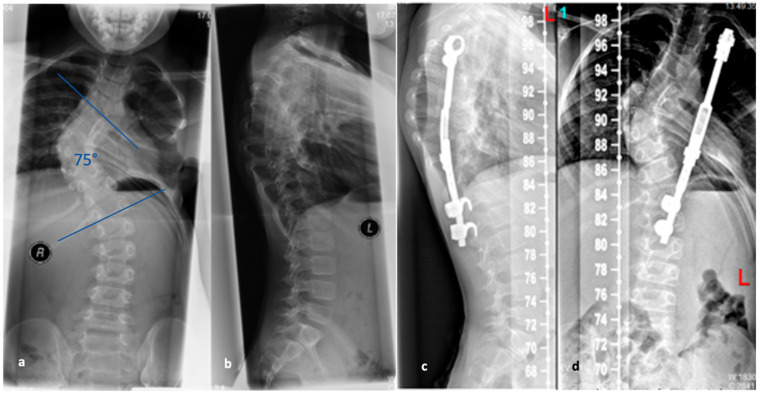
(**a**,**b**). Preoperative representation of the scoliosis and (**c**,**d**). Postoperative image following expansion thoracoplasty and implantation of the vertical expandable prosthetic titanium rib (VEPTR), attached proximal to the rib and distal to the laminae (reprinted with permission [10]).

The VEPTR system is a special type of scoliosis correction, and it is not considered as a classic growing rod. Originally, the system was used in children with congenital and highly progressive spinal deformities including unilateral brace formation and rib deformities with impending thoracic insufficiency syndrome [44]. The average age of 3.3 years at the time of surgery is lower for the VEPTR procedure in contrast to the TGR procedures. Currently, the main indication is thoracic scoliosis in congenital scoliosis with rib fusion, unilateral unsegmented braces, and contralateral hemivertebrae. This deformity is associated with severe thoracic asymmetry and markedly limited vital capacity of the lungs. The advantage of expansion thoracoplasty is that secondary correction of the spine can be achieved via correction of the rib deformity by rib osteotomies and VEPTR implantation without using a second approach in this area and thus increasing the risk for spontaneous fusions [45]. The VEPTR is a longitudinal, extendable titanium implant that is anchored to each of the caudal and cranial ribs using hooks of the concave hemithorax; see Figure 4. This “titanium rib” is also surgically expanded approximately every six months until growth is complete. Components may have to be replaced when the distraction distance is depleted. The main complication is cut-out or dislocation of the fixation to the ribs. By modifying the implant design, the complication rate could be reduced. In addition to intercostal connections from rib to rib, the VEPTR also offers the possibility of fixation to the laminae of the vertebral bodies or support on the pelvis.

#### 3.5.3. ApiFix^®^

The surgical procedure shown by Harrington, as already described, required regular surgical distraction. A system originating from Israel (ApiFix^®^) occupies a special position. In this system, a polyaxial screw is inserted on the concave side of the spinal deformity via a dorsal approach in the cranial and caudal end vertebrae of the concavity, which are connected via a distractable rod. The timing of use of the system is directed toward AIS. In a case collection of three adolescent patients, Floman et al. reported physiotherapeutic straightening of scoliosis using a ratchet mechanism by the patients themselves [46].

#### 3.5.4. Magnetically Controlled Growing Rods

**Figure 5:**
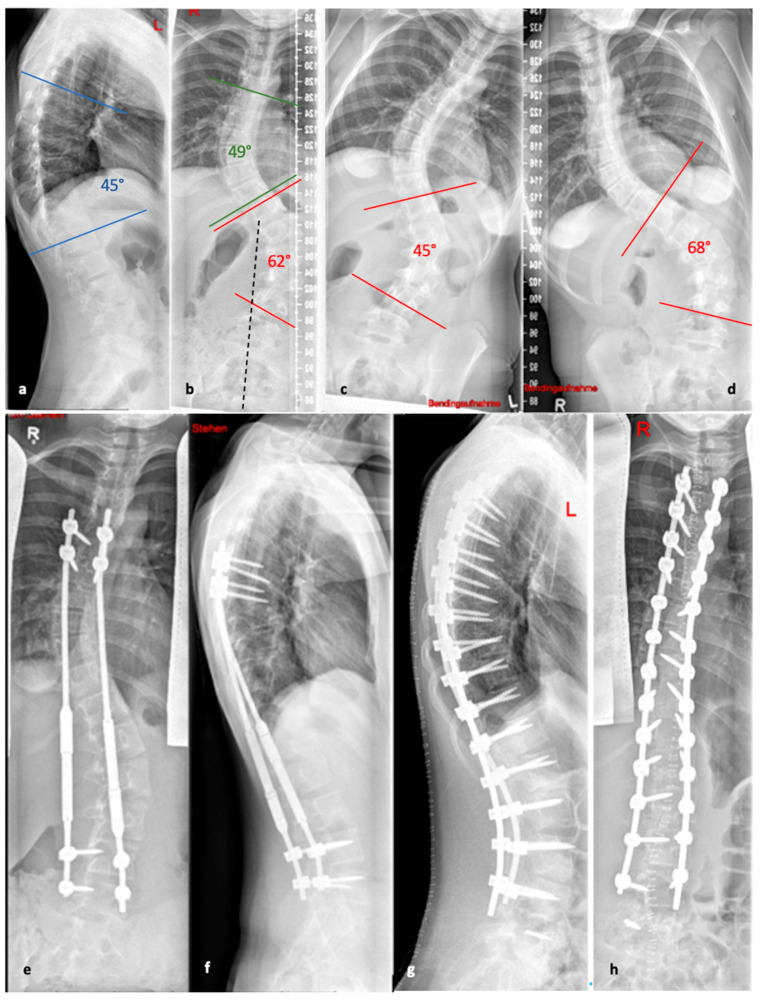
(**a**,**b**). Initial radiographs of a patient with adolescent idiopathic scoliosis. (**c**,**d**). Bending radiographs. (**e**,**f**). Postoperative image displaying implanted magnetically controlled growing rods as a dual rod system. (**g**,**h**). The patient was treated with a spondylodesis upon the completion of growth to maintain correction success (Reprinted with permission [10]).

To avoid the regular surgical lengthening procedure, MCGRs were developed and approved for the treatment of EOS in Europe in 2009. MCGRs are telescopically extendable distraction rods. They can be distracted non-invasively, on an outpatient basis, by externally magnetically controlled lengthening using electromagnets. Figure 5 illustrates the progression of treatment for a patient with AIS, starting from initial diagnosis, through the bending radiographs, to the postoperative phase with implanted MCGR, and finally, the maintenance phase involving spondylodesis.

### 3.6. Growth-Guiding Techniques

#### 3.6.1. Luqué Trolley Procedures

Luqué and Cardoso established a procedure that does not require surgical lengthening. For this purpose, the two distraction rods were implanted in a similar manner but are tied together with wire cerclages that provided a splint-like structure during growth along which the rods can distract [47]. However, this procedure shows comparably poorer results with higher rates of spontaneous fusion or implant failure and less correction of deformity [47].

#### 3.6.2. Shilla Technique

Unlike the previously described techniques, here, the spinal distraction is corrected via rods from the apex vertebra of the curvature. Instrumentation is first performed using pedicle screws in the region of the apex. Spondylodesis is performed and the rods are fixed in this short section. Cranial and caudal fixation is performed in more flexible areas of the curvature using polyaxial screws, which do not lock the rods but allow them to slide longitudinally within the screw head [48]. Aside from achieving favorable curve correction, this technique also eliminates the need for repeated open lengthening surgeries required by other methods. However, a high incidence of implant failure and wound complications necessitating revision surgery was observed [49].

### 3.7. Compression-Based Techniques

**Figure 6:**
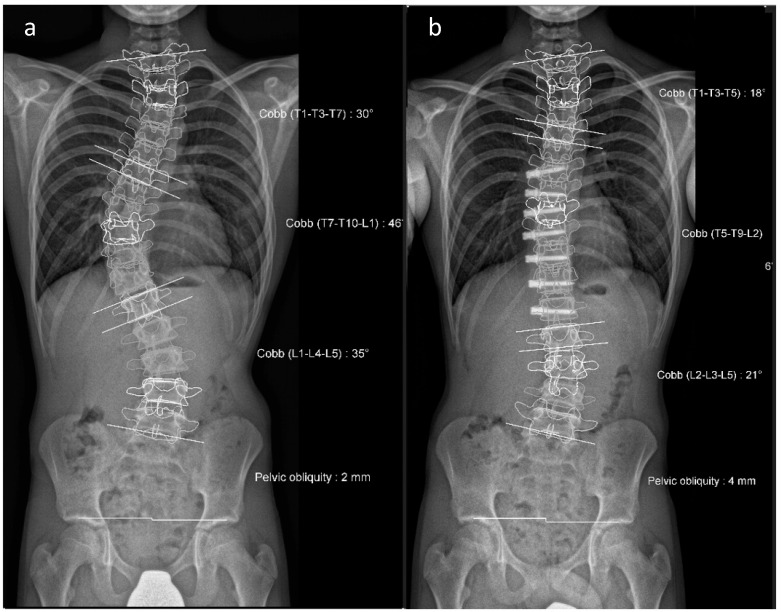
(**a**) Preoperative representation of the scoliosis. (**b**) Postoperative image following vertebral body tethering.

The compression-based procedures such as VBS or VBT are similar to temporary hemiepiphysiodesis, which inhibit the growth of the spine on the convex side by inserting the staples or ligaments and allow “catch-up” of the growth on the concave side of the curve; see Figure 6.

### 3.8. Results and Complications

#### 3.8.1. Results of Growing Rods

The use of growing rods has shown promising results in the treatment of EOS and AIS, but it is not without risks and complications. While studies have indicated that the use of dual rods may offer improved stability and a lower incidence of complications [50,51], besides higher complication rate for single rod constructions, there are no significant differences between single and dual growing rods with regard to deformity correction and spinal growth in the treatment of EOS [52]. Additionally, the use of TGR requires frequent surgeries for adjustment and lengthening, which comes with a significant risk of complications such as implant failure, infection, or wound-healing problems. On average, patients received 6.1 surgeries from primary implantation of the TGR to final spinal fusion [53]. The associated risk of complications during follow-up procedures is not insignificant and amounts to approximately 58%. Pedicle screw-based anchorage is commonly used now, along with hook or ligament systems depending on the anatomical conditions. Short-span fusion of two to three segments has been found effective to avoid implant loosening or fracture [49,50]. Adjacent segments should not be exposed subperiosteally to prevent spontaneous fusion [54]. The TGR technique has been shown to reduce curvature from 66 to 38° in 29 patients with a mean age of 6.7 years at the time of surgery [55]. Another study by Klemme et al. also reported similar success in curve reduction of about 50% [53]. While a non-invasive procedure (MCGR) has become established to perform the lengthening procedure at regular intervals, critical publications regarding metallosis and metalwork failures initially caused concern [56]. However, studies have shown that MCGR now shows comparable stability to TGR with a significant reduction in the complication rate [57].

Despite these improvements, the technical aspects of MCGR must be considered for the indication. The choice of the actuator position must be considered intraoperatively in order not to add to the resulting flat back formation (technically, it is not possible to bend the rod in the area of the distraction component, which may limit the use in kyphotic patients), and attention should be paid to the use of standard and offset rods [58,59]. Periodic lengthening is performed on an outpatient basis at four- to six-month intervals either with fixed distraction distances or individually adapted to the patient’s physiological growth [60,61]. Upon completion of spinal growth, the final step should be a definitive spinal fusion, involving the removal of the implanted distraction system and the correction of any remaining deformity. If the growing rods are removed without performing fusion, even after growth has stopped, there could be a recurrence of progression and a loss of the achieved correction [43,50].

#### 3.8.2. Complications of Growing Rods

Growing rods for growth guidance can lead to various complications, including infections, implant and fixation failure, and post-junctional kyphosis (PJK) or flat back problems [51]. Complication rates can be high, with some studies reporting up to 50% [62]. Age and the number of operations affect the risk, with a 24% increase per operation and a 13% decrease per year of life at primary operation [62]. Wound infections are more common in TGR than in MCGR (11.1% in TGR compared with 3.7% in MCGR) [63,64]. The crankshaft phenomenon may also occur during growth-guiding procedures [65]. PJK can occur at the cranial end of the instrumentation, and its rate is around 29% for both TGR and MCGR with higher risk for hyperkyphosis [66]. Despite the significant complications, early surgical treatment of scoliosis is essential for patients requiring therapy until growth is complete [67].

While growing rods offer a viable treatment option for scoliosis, it is important to carefully consider the risks and benefits before deciding the indication for surgery. Close monitoring and prompt treatment of complications are crucial in ensuring the best possible outcome for patients with scoliosis.

#### 3.8.3. Results of Vertebral Body Tethering

VBT is a recently (FDA approval in August 2019) developed method for correcting the curvature of the spine in patients with AIS. This technique involves applying compressive force to the convex side of the curve, which helps to regulate the growth of the spine. Although VBT is similar to VBS, it has been found to be more effective [68]. A recent systematic conducted by Zhang et al. showed that VBT was both safe and effective [68]. Most of the studies indicated a significant improvement in the major curve correction when comparing preoperative versus postoperative and final follow-up results (average preoperative main curve Cobb angle: 40–56° vs. average postoperative main curve Cobb angle: 14–38°, with a correction rate of 15.6–69.4% vs. average main Cobb angle at the final follow-up: −3 to 38°, with a correction rate of 15.6–106.5%) [68]. Confirmed or suspected broken tether (21.3%), pulmonary and respiratory complications (6.9%), and overcorrection (4.2%) were found to be the most frequent complications associated with VBT with a revision rate of 13.1% [68]. In contrast, a meta-analysis by Marshal et al. revealed that the most common complications included reoperation (10.1%), overcorrection (8.0%), and tether breakage (5.9%) [69]. A large proportion of VBT patients managed to avoid spinal fusion, with only 4.7% requiring conversion to posterior spinal fusion due to unsuccessful tethering [70].

There is a lack of published studies on the outcomes and complications of VBT, resulting in a considerable variation in the reported rate of complications. As a result, the true complication rate of VBT is yet to be determined. Additionally, there is a lack of long-term studies related to the rates of complications and reoperation.

### 3.9. Halo Gravity Traction

Preoperative halo-gravity traction (HGT) has been extensively employed in the clinical treatment of severe deformities, including instances of scoliosis characterized by a Cobb angle > 90° to 100° [71]. Initially reported by O’Brien et al. in 1971 for the treatment of scoliosis [72], this technique involves the application of a surgically fixed halo and can be performed as either halo-gravity traction or halo-pelvic traction [73,74]. Traction enables a gradual correction of the curve in the frontal, sagittal, or axial plane while facilitating continuous and conscious neurological monitoring. HGT can be employed to achieve partial correction, leading to a reduction in surgical complexity and a decreased risk of neurological injury associated with excessive operative correction [75].

Moreover, HGT has the potential to enhance flexibility and final correction when combined with surgical release. Preoperative HGT has also demonstrated benefits in improving pulmonary function and nutritional status, thereby boosting patients’ surgical tolerance. A recent meta-analysis by Wang et al. [76] highlighted a median correction of the Cobb angle by 28° and a correction of thoracic kyphosis by 26°, with significant improvements in pulmonary function and nutritional status. In this meta-analysis, the mean duration of halo traction was 50 days, ranging from 28 to 79 days [76].

However, the use of HGT is not without its contraindications or complications. Certain conditions, such as cranial malformations that prevent proper pin placement, osteogenesis imperfecta (considered a relative contraindication), and osteoporosis (addressed by using a higher number of pins tightened at a lower torque) may prohibit its use. Absolute contraindications include the presence of intra or extradural growths, medullary canal stenosis, and neurological deficits [73]. Common complications, such as cervical and back pain, headache, and vertigo, often result from high traction force and can usually be relieved by a reduction in weight. Pin complications, including pin pain, infections, and displacement, as well as more severe complications such as deep infections and neurological issues, are relatively rare but must be closely monitored [73]. Despite these challenges, a careful monitoring of patients can ensure most complications are addressed effectively with minimal adverse outcomes [74].

## 4. Scheuermann’s Disease

The most common growth disorder that affects the sagittal profile of the spine is SD, which is also known today as juvenile osteochondritis deformans of the spine. SD was first described in 1921 as a rigid developmental thoracic kyphosis by the Danish radiologist and orthopedist Holger Werfel Scheuermann (1877–1960). Radiologically, a ventral flattening of the vertebral bodies with “wedge-shaped” vertebral bodies as well as rigidity of the spine was detected in the lateral X-ray [77].

Generally, SD is defined as a hyperkyphosis with a Cobb angle of the thoracic kyphosis >45° and tends to manifest in the thoracic spine, where the prognosis is more favorable than in cases of thoracolumbar deformity. The latter is typically more painful and has a higher likelihood of progression [26]. This is caused by wedge-shaped vertebral bodies, along with growth disturbances of the endplates (known as Schmorl’s nodes) [78]. At least three adjacent vertebrae are affected with a wedging angle of at least 5° each [79]. Additionally, there is often elongation of the sagittal vertebral body diameter and shortened ischiocrural muscles. Nevertheless, patients usually do not show any significant neurological symptoms. The displacement of the upper body center of gravity forward usually leads to lumbar pain in patients as the leading symptom.

The exact cause of SD is not known. However, a familial cluster with autosomal-dominant inheritance is reported [80]. In the study by Damborg et al. between 1931 and 1982 in Denmark with 35,000 twins, a prevalence of 2.8% and a heritability of 74% were described [81].

**Figure 7:**
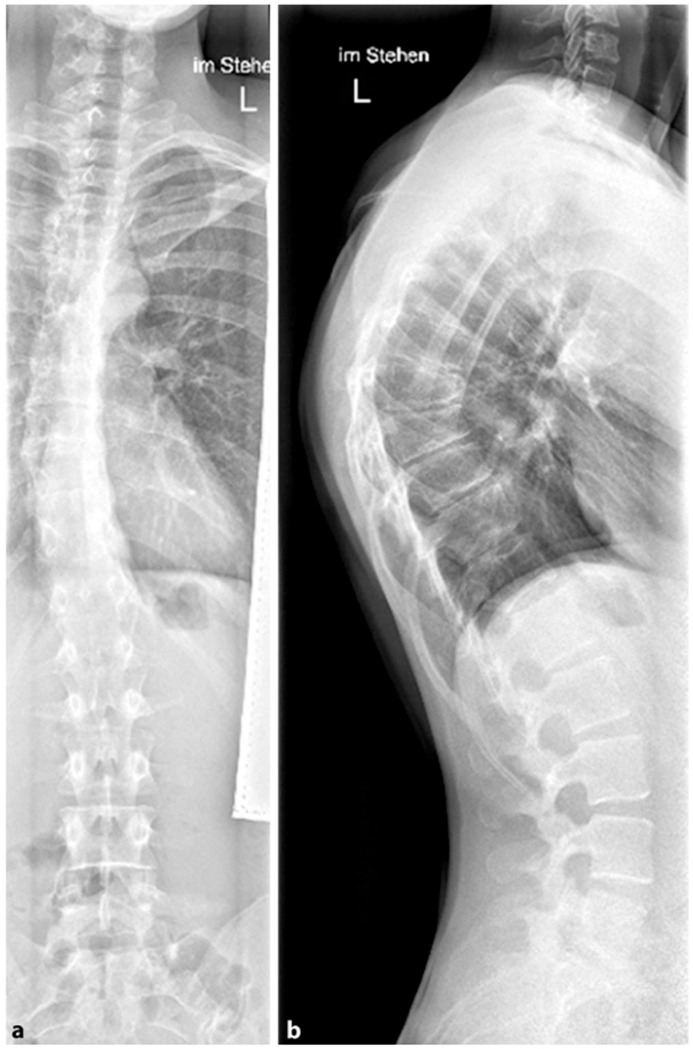
Classic Scheuermann’s disease of the thoracic spine with mild thoracic scoliosis (reprinted with permission [23]). (**a**)—posterior-anterior view, (**b**)—lateral view.

The prevalence of SD is 4–6% in the general population and 1–8% in the group of adolescents. The sex ratio describes a more frequent occurrence in males (male:female = 2:1) [6]. Clinical and radiological changes defining the age of onset can occur from the age of 10–12 years [26] (Figure 7). The typical changes in the shape of the vertebral bodies are generally stabilized after the end of growth. However, a secondary deterioration of the hyperkyphosis is possible after the end of growth. In addition to the structural kyphosis in SD, there are various differential diagnoses for hyperkyphosis (Table 4).

### 4.1. Prognosis

The prognosis of SD is mostly good. Murray et al. described how the curvatures below 85° do not impair pulmonary function, while kyphotic curvatures over 100° and with an apex in the uppermost eight thoracic vertebral bodies exhibit a significant restrictive ventilation disorder [82]. Ragborg et al. observed a significant reduction in quality of life compared to the general population in a cohort with a 39-year follow-up [83]. Garrido et al. described a worsening of the kyphosis angle of 0.45° annually and a deterioration of the Oswestry Disability Index (ODI) compared to the general population in a cohort of untreated SD patients with a 27-year follow-up [84].

### 4.2. Diagnostics

**Figure 8:**
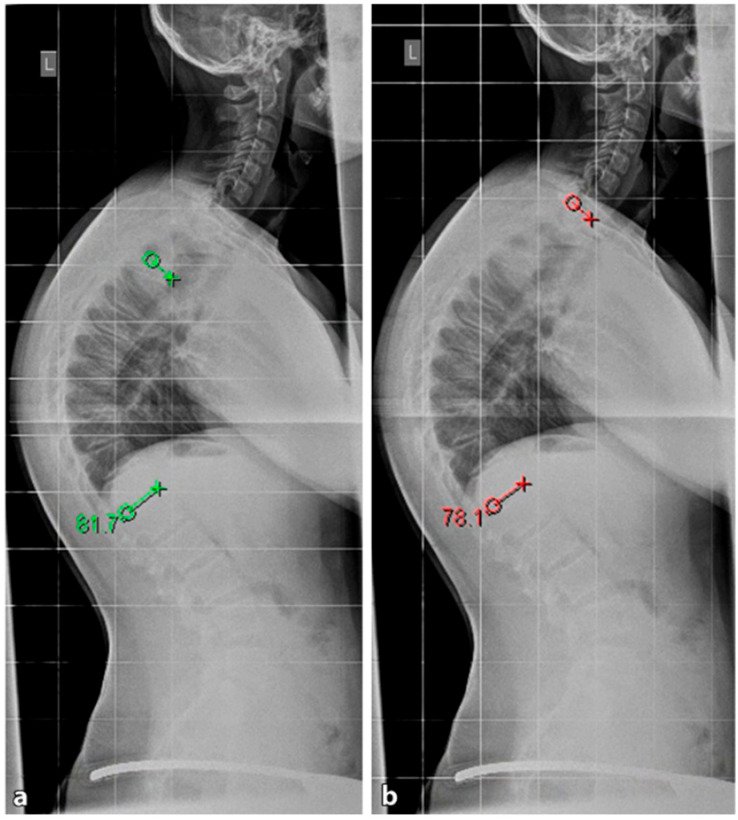
Classic Scheuermann’s disease of the thoracic spine. (**a**). Green—Stagnara angle (T4–T12), (**b**). Red—Cobb angle (T1–T12) (reprinted with permission [23]).

Although initially described by Sorensen, the definition of SD has been definitively established by subsequent work by Edgren et al. and Blumenthal et al., with SD typically manifesting mostly in the thoracic spine (type I) and less commonly in the lumbar spine (type II) [79,85,86]; see Figure 7. The measurement of thoracic kyphosis according to Cobb (Th1–Th12) is sometimes difficult on conventional X-rays due to the humeral heads, so the Stagnara angle (Th4–Th12) is a reliable alternative (Figure 8). In children with SD, back pain and a positive family history are common, and clinical examination typically reveals a gibbus and a flexible deformity. In addition to a detailed medical history, a thorough physical examination is of utmost importance. The various signs and findings typical of SD are listed in Table 2.

#### 4.2.1. X-ray

A standing anterior–posterior and lateral spinal radiograph is part of the standard imaging. Stereoradiographic imaging can be used alternatively to conventional radiography to create a three-dimensional reconstruction of a biplanar spinal X-ray with significantly less radiation exposure (EOS™) [31]. In addition to the representation of the kyphosis in the lateral view, these images also show the extent of curvature in the frontal plane according to Cobb, the curvature pattern, the sagittal profile or sagittal balance, and the apical vertebra. An assessment of spinal flexibility can be made using a clinical examination or a hyperextension lateral radiograph. The various features can be observed in radiological diagnostics, as shown in Figure 7. In up to 50% of cases, scoliosis and spondylolysis are accompanying pathologies [87,88].

#### 4.2.2. Magnetic Resonance Imaging

**Figure 9:**
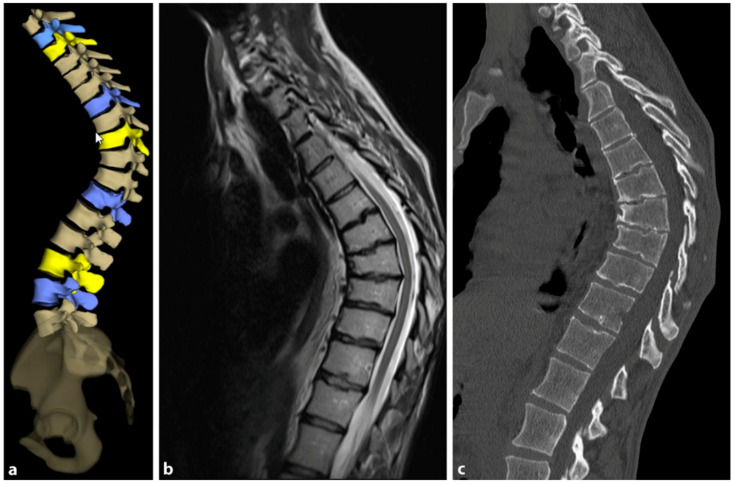
(**a**) Stereoradiographic imaging, (**b**) magnetic resonance imaging (MRI) and (**c**) computer tomography (CT) images of thoracic Scheuermann’s disease (reprinted with permission [23]).

The need for MRI diagnosis has been demonstrated in several studies. In a recent study, the prevalence of syringomyelia was found to be 5.8% in patients with SD [89]. MRI is an obligatory preoperative preparation to exclude myelon compression, thoracic disc herniation, or spinal canal stenosis. Figure 9 provides a comparative visualization of thoracic SD captured through different imaging techniques: stereoradiography, MRI, and computer tomography (CT).

### 4.3. Conservative Therapy

**Figure 10:**
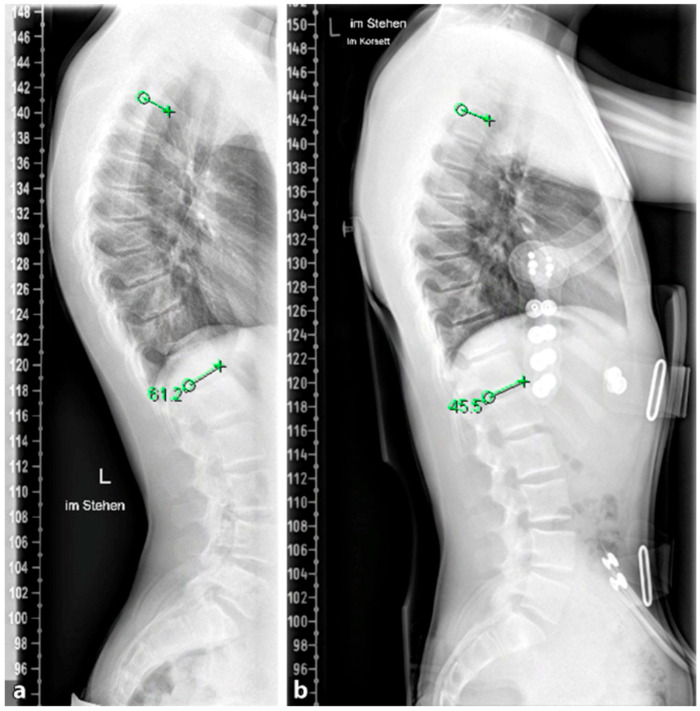
(**a**) X-ray of the full spine lateral view without brace, (**b**) Correction of mild hyperkyphosis in reclination brace (from 61.2° to 45.5°) (reprinted with permission [23]).

The goal of orthopedic treatment is to relieve pain by reducing pressure on the anterior aspect of the vertebral endplates. In addition, it can facilitate the healing of certain local lesions. The conservative treatment of SD depends on the degree of curvature. Curves under 60° are well compensable with spinal physiotherapy and other supportive measures, while curves of 60–80° respond well to brace therapy in most cases [26]; see Figure 10. For less pronounced forms (<60°), physiotherapy with extension of the spine and retraction of the scapulae and rehabilitation according to Katharina Schroth, which is also successfully applied in milder forms of scoliosis, provides good pain relief and in some cases even an improvement in kyphosis [90,91,92]. Brace therapy is well suited for curves >60°. Various types of braces show good results. The principle of correction is based on a dorsal pad with two thoracic pads that achieve a de-kyphosis of the thoracic spine.

### 4.4. Surgical Therapy

While the majority of patients with SD generally show a favorable response to non-surgical interventions and experience slow or no progression of their kyphosis, in cases of particularly severe progression or when conservative treatment proves ineffective, surgical intervention after the completion of growth may be necessary [93,94,95]. Absolute indications for surgical treatment are neurological deficits or signs of thoracic myelopathy, whereas relative indications exist for patients with a kyphosis angle above 75° thoracic or 65° at the thoracolumbar junction, an unacceptable deformity (e.g., kyphosis of the upper lumbar spine) as well as severe pain [96,97].

**Figure 11:**
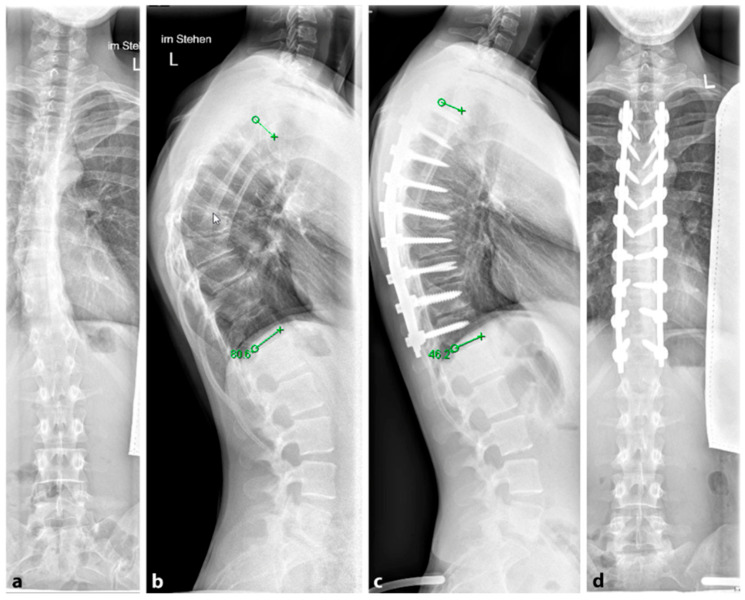
(**a**,**b**). Preoperative and (**c**,**d**). postoperative radiographs after corrective spondylodesis from T4 to T12 with ventral release, correcting the Stagnara angle from 80 to 46 degrees (reprinted with permission [23]).

Several surgical techniques have been proposed to address SD, all of which involve three key steps: release of spinal structures, correction of the kyphosis (aiming for at least 50% correction), and spondylodesis with instrumentation [96]; see Figure 11. Some techniques include an anterior release to facilitate posterior curve correction, but the potential benefits of this approach remain uncertain, and it may lead to a higher occurrence of unfavorable effects such as higher complications rates, blood loss and operation time [98,99,100]. Earlier studies have shown favorable outcomes in terms of pain relief and spinal deformity correction following surgical intervention for SD with the most common complications reported being neurological (such as paraplegia), infectious, and respiratory in nature [93,95]. For rigid curvatures, anterior release followed by dorsal instrumentation and spondylodesis was the method of choice until about 20 years ago. However, in several studies, dorsal instrumentation with osteotomies and shortening of the dorsal column achieved comparable results to anterior–posterior procedures [101,102]. Lee et al. evaluated 17 studies and a total of 1147 patients in a meta-analysis. Here, the correction of the kyphotic malalignment between dorsal instrumentation with osteotomies and anterior–posterior spondylodesis was comparable [103]. Despite this, anterior release, fusion and posterior spinal fusion experienced significantly more complications than the posterior spinal fusion alone [101]. Therefore, it is not recommended to subject the patient to additional surgery (i.e., anterior release and fusion). A single posterior approach is adequate to achieve sagittal correction with a balanced spine and fewer associated complications.

The rationale of both procedures and respective advantages and disadvantages can be found in Table 5.

#### 4.4.1. Selection of the Instrumentation Range

**Figure 12:**
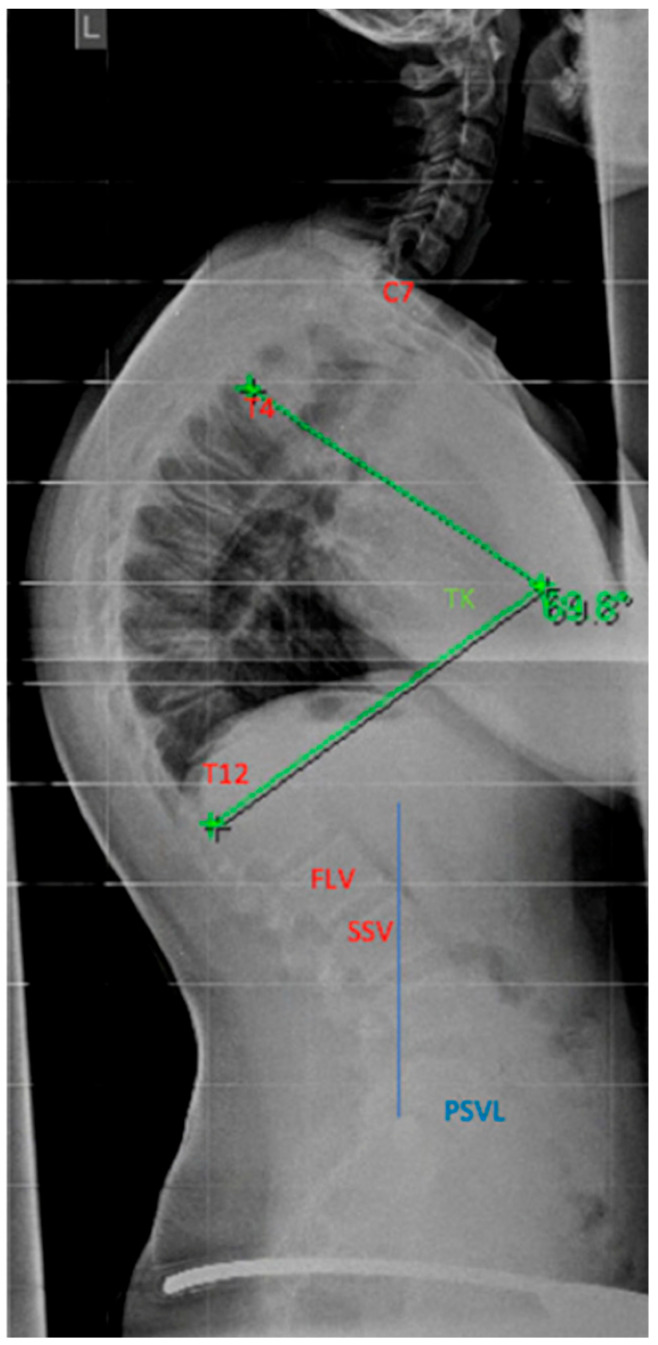
FLV—first lordotic vertebral body, SSV—sagittal stable vertebral body, PSVL—posterior sagittal vertical line, TK—thoracic kyphosis (reprinted with permission [23]).

The determination of the instrumentation range is certainly dependent on the chosen surgical procedure. The upper instrumented vertebra (UIV) is usually the proximal vertebra that still belongs to the curvature [104]. The selection of the lowest instrumented vertebra (LIV) can be the first lordotic vertebra (FLV) or the sagittal stable vertebra (SSV) according to Cho et al. [105]; see Figure 12. However, in the meta-analysis by Gong et al., instrumentation of the SSV proved to be superior to the FLV [106]. In this meta-analysis, the incidence of distal junctional kyphosis (DJK) in SD was reported to be 20.8%, and of these cases, 27.8% of patients had to be revised; 5.9% of the SSV cohort and 43.6% of the FLV cohort developed DJK [106].

#### 4.4.2. Reduction Techniques

The reduction techniques can be chosen depending on the procedure, the experience of the surgeon, the location of the apical vertebra, and the quality of the bony conditions. Uniplanar, monoaxial, or reduction pedicle screws can be used for distraction, compression, extension, or flexion [107]. To avoid PJK, a correction of less than 50% of the original curve should be aimed for.

#### 4.4.3. Osteotomies

The most commonly performed osteotomies in the surgical treatment of SD are partial facetectomies and the apical Ponte osteotomy (osteotomy according to Schwab Grade II) [108]. Its low-risk profile and quick application allow for good correction of the deformity. In fixed or rigid kyphotic malalignment, pedicle subtraction osteotomy (PSO) (osteotomy according to Schwab Grade III or IV) is possible [108].

#### 4.4.4. Complications

Complications in the surgical treatment of SD are 3.9 times more likely than in the surgical treatment of idiopathic scoliosis [109]. An indispensable component in the surgical correction of hyperkyphosis is intraoperative neuromonitoring. An intraoperative signal loss is much more common during the correction of hyperkyphosis or kyphoscoliosis than during the correction of scoliosis. However, the application of the different possibilities (motor-evoked potentials, somatosensory-evoked potentials) requires fundamental knowledge of neurophysiology.

## 5. Conclusions

The management of scoliosis in young children is a complex process that requires a tailored approach based on various factors such as the patient’s etiology, curve pattern, skeletal maturity, and co-morbidities. While there is no one-size-fits-all solution for every patient, it is widely accepted that surgical intervention, including growth-friendly techniques, should be delayed for as long as possible. For children, there are various implant options available to spinal deformities. Growth-modulated constructs, such as growing rods, vertical expandable prosthetic titanium ribs, and vertebral body tethering, are commonly used to correct the scoliosis dynamically as the patient matures if significant growth remains. Vertebral body tethering is a novel technique that offers the advantage of correcting idiopathic deformities while preserving motion, particularly in juvenile cases with moderate curvature. However, to better understand the complications associated with this technique and how to prevent them, further long-term studies are necessary. 

In children with depleted growth potential or those with SD, static fixation methods are available. These methods often share implant types and techniques with dynamic fixation options. In conclusion, while several surgical procedures have shown promising results in the treatment of scoliosis and Scheuermann’s disease, there is a need for further studies to establish long-term efficacy, safety, and the optimal parameters for each application to ensure the best patient outcomes.

## Figures and Tables

**Table 1:** Differential diagnoses of scoliosis [10].

Scoliosis	
Functional scoliosis	Compared to structural scoliosis, functional scoliosis is merely a dynamic lateral bending in the frontal plane without a rotational component, which does not show any changes in the shape or structure of the spine or vertebral bodies on radiological imaging. Functional scoliosis is always reversible and indicates a disturbance in postural or movement symmetry (e.g., leg length discrepancy).
Functional scoliosis in infants	Functional scoliosis of infants is a special form of functional scoliosis. It occurs a few months after birth and shows a multisegmental, C-shaped, left-convex lateral bending in the thoracolumbar region of the spine. Spontaneous remission is very common.

**Table 2:** Diagnostic features of scoliosis and Scheuermann’s disease [10,23].

Diagnostic Features	Scoliosis	Scheuermann’s Disease
Patient history	Disorders (genetic, syndromes, neuromuscular diseases, secondary scoliosis) Previous surgeries Family history Symptoms (painless?) Spinal trauma Radiotherapy	Disorders (genetic, syndromes, neuromuscular diseases) Previous surgeries Family history Symptoms (pain?) Spinal trauma Radiotherapy
Clinical examination	Asymmetrical waist triangles Rib rotational deformities (rib prominence/hump): scoliometer test >5° Lumbar prominence Thoracic deformities (asymmetrical pectus excavatum/carinatum) Leg length inequity Pelvic obliquity Shoulder height difference Axial deviation of the spine from the perpendicular with lateral overhang of the trunk/trunk shift Flat back due to reduction in thoracic kyphosis and lumbar lordosis	Obesity Thoracic hyperkyphosis Lumbar hyperlordosis Thoracic deformities Gibbus Contracture of ischiocrural muscles
Radiological findings	X-ray (posterior–anterior and lateral view), in the standing position (determination of the lateral inclination in the frontal plane: Cobb angle) Alternative: EOS™ (three-dimensional reconstruction of the spine	X-ray (posterior–anterior and lateral view), in the standing position (determination of the deformity in the sagittal plane: Stagnara angle Th4–Th12) Wedge-shaped vertebral formation Alternative: EOS™ (three-dimensional reconstruction of the spine MRI: Schmorl’s nodes

MRI—magnetic resonance imaging.

**Table 3:** Summary of different surgical procedures for the treatment of scoliosis.

Features	Distraction-Based Techniques	Compression-Based Techniques	Growth-Guiding Techniques
Systems	Traditional growing rods Magnetic-controlled growing rods Vertical Expandable Prosthetic Titanium Rib (VEPTR) ApiFix^®^ Harrington rods	Vertebral body stapling (VBS) Vertebral body tethering (VBT)	Shilla technique Luqué trolley technique
Indication	Early onset scoliosis Thoracic Cobb angle >50° Lumbar Cobb angle >40–45° Sanders ≤3	Adult idiopathic scoliosis <60° Bending <25° Kyphosis <40° Sanders ≤5	Early onset scoliosis Thoracic Cobb angle >50° Lumbar Cobb angle >40–45° Sanders ≤3
Advantages	Fusionless procedure Preservation of residual growth capacity of the spine No consecutive revision surgery for correction (except for traditional growing rods, Harrington rod, VEPTR) Preservation of the mobility of the spine (VBT)		
Dis- advantages	Consecutive revision surgery for correction for traditional growing rods, Harrington rod, VEPTR) Proximal junctional kyphosis Spontaneous fusion Magnetic-controlled growing rods: contraindicated in patients who require repetitive MRI	Limited indication: not suitable for secondary or early onset scoliosis Less severe scoliosis No valid long-term results	No valid long-term results

MRI—magnetic resonance imaging.

**Table 4:** Differential diagnosis of Scheuermann’s disease [23].

Scheuermann’s Disease	
Postural kyphosis	Compared to structural kyphosis, postural kyphosis represents a dynamic bending in the sagittal plane that does not show any changes in the shape or structure of the vertebral bodies on radiological imaging. Postural kyphosis is usually reversible and indicates a postural or musculoskeletal disorder (e.g., shortening of the ischiocrural muscles).
Multisegmental kyphosis (ankylosing spondylitis—Bechterew’s disease)	This is a kyphotic deformity caused by a dynamic change in vertebral body and disc anatomy or ligamentous anatomy (ankylosing spondylitis) or a combination of both structures. More rarely, an underlying neuromuscular disease is the cause of kyphosis.
Angular kyphosis	Various clinical conditions can cause of this type of kyphosis including the various forms of congenital kyphosis (dorsal wedge-shaped vertebra, ventral block formation), bacterial or tuberculous spondylodiscitis, post-laminectomy syndrome, neoplasia, osteoporotic fractures.

**Table 5:** Description of the technique, advantages and disadvantages of different surgical approaches for Scheuermann’s disease [23].

Procedure	Technique	Advantages	Disadvantages
Anterior release and dorsal instrumentation	Involving approximately seven segments around the apex Standard thoracotomy or thoraco-abdominal approach Complete discectomy and release of the anterior longitudinal ligament at each segment Single-stage or two-stage surgery Posterior construct: pedicle screws, hooks/claws, hybrid (i.e., a combination of pedicle screws and hooks/claws), Optional: alternate segment pedicle screws	Better correction of kyphosis Shorter range of fusion/instrumentation	Dual surgical approaches Higher morbidity Higher complication rates Neurological deficits Surgical site infections Respiratory deficits Higher blood loss
Dorsal instrumentation with osteotomies	Posterior column osteotomies: partial facetectomies, apical Ponte osteotomy, and multiple Chevron osteotomies Instrumentation and fusion Hooks or pedicle screws Reduction technique: rod cantilevering and sequential segmental compression Decortication of posterior elements Use of autologous bone grafts for arthrodesis	Single surgical approach Shorter operation time Faster rehabilitation	Inferior correction without osteotomies Higher demands in instrumentation technology

## Data Availability

Not applicable.
